# Seroprevalence of SARS-CoV-2 antibodies in low-income university students

**DOI:** 10.17179/excli2021-3459

**Published:** 2021-02-09

**Authors:** Adriano Antunes de Souza Araújo, Lucindo José Quintans-Júnior, Dulce Marta Schimieguel, Cristiane Bani Corrêa, Tatiana Rodrigues de Moura, Rafael Ciro Marques Cavalcante, Renata Grespan, Daniele de Vasconcelos Cerqueira-Meneses, José Antônio Barreto-Alves, Paulo Ricardo Martins-Filho

**Affiliations:** 1 Department of Pharmacy, Federal University of Sergipe, São Cristóvão, Sergipe, Brazil; 2 Health Sciences Graduate Program, Federal University of Sergipe, Aracaju, Sergipe, Brazil; 3 Graduate Program in Pharmaceutical Sciences, Federal University of Sergipe, São Cristóvão, Sergipe, Brazil; 4 Department of Physiology, Federal University of Sergipe, São Cristóvão, Sergipe, Brazil; 5 Department of Morphology, Federal University of Sergipe, São Cristóvão, Sergipe, Brazil; 6 Department of Pharmacy, Federal University of Sergipe, Lagarto, Sergipe, Brazil; 7 Graduate Program in Physiological Sciences, Federal University of Sergipe, São Cristóvão, Sergipe, Brazil; 8 Graduate Program in Nursing, Federal University of Sergipe, Aracaju, Sergipe, Brazil; 9 Investigative Pathology Laboratory, Federal University of Sergipe, Aracaju, Sergipe, Brazil

## ⁯⁯

***Dear Editor,***

The COVID-19 pandemic has brought about major educational, social, and economic changes around the world. Even as cases of COVID-19 still surge in many countries, the debate over reopening universities remains at the forefront of the present crisis (Tilley et al., 2020[5]). However, in high-poverty settings, many students live in conditions of social and economic vulnerability, which increases the risk of SARS-CoV-2 infection and worse outcomes associated with disease (Martins-Filho et al., 2021[4]). Studies published to date on the occurrence of COVID-19 in university students especially in middle- and low-income countries are scarce. We investigated the seroprevalence of SARS-CoV-2 antibodies in low-income university students at a public higher education institution in Northeastern Brazil. 

This cross-sectional study included students registered with the *Programa de Auxílio Moradia e Residência Universitária (PAMRU - *the Housing and University Residency Assistance Program) at the Federal University of Sergipe (UFS), Sergipe State, Northeast Brazil. UFS was founded on May 15, 1968, and is the only public university in Sergipe State, with 26 407 undergraduate students enrolled in 118 on-site courses spread over five campuses (São Cristóvão, Itabaiana, Lagarto, Laranjeiras and Nossa Senhora da Glória). According to the UFS Student Registration Questionnaire, 49.4 % of students do not have any type of income, while 33.9 % receive up to one Brazilian minimum wage per month. Of the total enrolled students, 1450 (5.5 %) are registered with PAMRU, which includes students from other states of the Federation, or those who live outside the urban area of the UFS campus at which they study. Assistance with housing is granted to selected students following an application process based on a socioeconomic evaluation. All students registered with PAMRU were invited to participate in this study through an invitation letter sent by the Integrated System for the Management of Academic Activities (SIGAA). 

The study was conducted from September 15 to October 6, 2020 at all campuses of the university and consisted of a structured interview about clinical and demographic issues and aseptic collection of venous blood for the detection of IgM and IgG antibodies to SARS-CoV-2. For the serological analysis, an immunoassay was performed using fluorescent immunoassay (FIA) technology (iChroma II, BioSys + Kovalent). A result was considered positive if the automated reader had a reading ≥1.1. The sensitivity and specificity of this immunoassay are 95.8 % and 97.0 %, respectively, according to the manufacturer when evaluated in 46 patients positive for SARS-CoV-2 and 131 negative controls. The main outcome of the present study was the seroprevalence of SARS-CoV-2 antibodies, expressed as the proportion of students who tested positive in the serological test. Seroprevalence with 95 % confidence interval (CI) was calculated using the Wilson procedure. 

Of the 1450 students registered with PAMRU, 276 (19.0 %) participated in the study: 150 (54.4 %) female students and 126 (45.6 %) male students. Median age was 22.0 years (IQR 21.0 - 24.0) and the median number of residents per university residence was 4.0 students (IQR 2.0 - 5.0). Sixty-two students had antibodies to SARS-CoV-2 and the overall seroprevalence was 22.5 % (95 % CI 17.9 - 27.7). Table 1(Tab. 1) shows the proportion of students examined on each university campus and the estimated seroprevalence. Of the 62 students with antibodies to SARS-CoV-2, 10 (16.1 %) had some pre-existing health condition including asthma or rhinitis (n=5), obesity (n=3), and cardiovascular disease (n=2). Most of the students with antibodies to SARS-CoV-2 (n = 42, 67.7 %) reported the occurrence of symptoms associated with COVID-19, such as headache (n=33; 53.2 %), loss of taste and/or smell (n=26, 41.9 %), fever (n=25; 40.3 %), cough (n=22, 35.5 %) and coryza (n=21, 33.9 %). Less common symptoms include musculoskeletal pain (n=12, 19.4 %), diarrhea (n=8, 12.9 %), dyspnea (n=6, 9.7 %), and vomiting or nausea (n=5, 8.1 %). 

Brazil is facing a major dilemma as to when and how to return to in-person academic activities even with the national vaccination plan having started with health professionals, institutionalized elderly, and indigenous people. On December 7, 2020, a decree was published in an extra edition of the Official Gazette of the Federal Government determining the return to face-to-face classes at public and private universities from March 1, 2021. However, in countries like Brazil, the number of low-income students enrolled in public universities is significant and many of them live in conditions of extreme social vulnerability and often need financial assistance or housing. In addition, the risk of COVID-19 transmission is expected to be higher in university residences, where the density of students and the potential for interaction are greater. Thus, the assessment of the seroprevalence of antibodies to SARS-CoV-2 is essential to the development of institutional policies to monitor the contamination profile of this population.

In the present study, we found a seroprevalence of 22.5 % (95 % CI 17.9-27.7) for SARS-CoV-2 antibodies among the students, with rates ranging from 16.0 % (95 % CI 10.6-23.4) to 28.6 % (95 % CI 8.2-64.1) depending on the evaluated campus. In a study carried out with university students in Los Angeles (USA) (Tilley et al., 2020[5]) using ELISA immunoassay in April and May 2020, the estimated prevalence for SARS-CoV-2 antibodies was 4.0 %, but only 9.1 % of the evaluated students lived in university residences. The differences found between the two studies may, in part, reflect the different periods of the pandemic at which the data were collected. Moreover, the interruption of classroom activities in Brazil and the return of the students to their homes probably resulted in contact with people infected with SARS-CoV-2 in the community, contributing to this high prevalence rate. 

Some important data found in this study included the large proportion of students tested positive for COVID-19 that reported symptoms of loss of taste or smell but did not have a history of respiratory symptoms. Less than 40 % of students with detectable antibodies reported cough or runny nose, and only 9.7 % had respiratory distress. In contrast to studies that indicate a higher likelihood of asymptomatic infection in young people (Kronbichler et al., 2020[1]), we observed a higher proportion (67.7 %) of symptomatic students among those diagnosed with COVID-19. However, it is possible that these data were influenced by the motivation or interest of individuals to participate in the study, especially among students with a history of symptoms suggestive of COVID-19.

At the time of writing this manuscript, academic activities at UFS were being carried out remotely because of the ongoing active community transmission of SARS-CoV-2 in Sergipe State. Before resuming on-site activities, an organized public health plan needs to be implemented considering the large number of students, teachers, and employees of the institution. The resumption of classroom activities without a well-defined strategy can lead to outbreaks of the disease in the university environment (Marris, 2020[2]). The reopening of higher education institutions will depend on the ability to mitigate the spread of the disease through continuous physical distancing measures, environmental measures, the promotion of behaviors that reduce transmission, contact tracking and access to tests (Tilley et al., 2020[5]). Moreover, it has been suggested that the implementation of structured surveillance programs in high-risk environments such as university residences is a fundamental strategy for controlling the transmission of the disease in the academic community as face-to-face activities are resumed. In Sergipe State, it has been found an association between COVID-19 mortality and poverty reinforcing the need of specific strategies for the most vulnerable people (Martins-Filho et al., 2020[3]).

The present study shows a high seroprevalence of SARS-CoV-2 antibodies in low-income university students even with the interruption of on-site academic activities and the return of students to their homes. These results may contribute to a better understanding of infection rates in this population and the development of a well-structured plan for the return of face-to-face activities.

## Acknowledgements

To all health professionals who are facing the COVID-19 pandemic. This study is part of the EpiSERGIPE project which is supported by grant SES/FAPESE/UFS 001/2020. The funding source had no role in the design and conduct of the study; collection, management, analysis, and interpretation of the data; preparation, review, or approval of the manuscript; and the decision to submit the manuscript for publication. We dedicate this article to all the doctors and frontline health workers and other staff for their dedication in the fight against COVID-19.

## Ethical aspects

This study was approved by the Ethics Committee of the Federal University of Sergipe (protocol number 34240620.7.0000. 5546). Written informed consent was obtained from all study participants.

## Figures and Tables

**Table 1:**
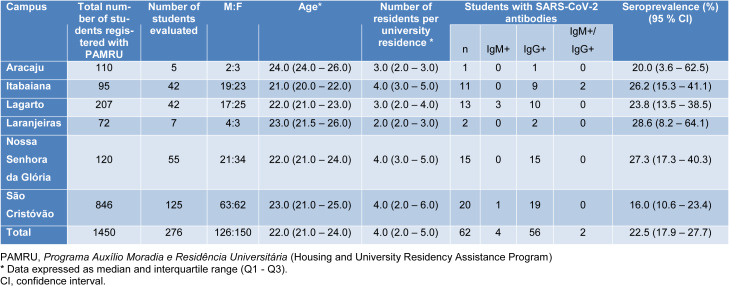
General characteristics of the students receiving housing assistance by university campus and seroprevalence of antibodies to SARS-CoV-2.
